# Post-placement Lead Deformation Secondary to Cerebrospinal Fluid Loss in Transventricular Trajectory During Responsive Neurostimulation Surgery

**DOI:** 10.7759/cureus.6823

**Published:** 2020-01-30

**Authors:** Panagiotis Kerezoudis, Elaine Wirrell, Kai Miller

**Affiliations:** 1 Neurological Surgery, Mayo Clinic, Rochester, USA; 2 Child and Adolescent Neurology, Mayo Clinic, Rochester, USA

**Keywords:** responsive neurostimulation, lead, deformation, cerebrospinal fluid, trajectory

## Abstract

Responsive nerve stimulation (RNS) represents a safe and effective treatment option for patients with medically refractory temporal lobe epilepsy. In cases of long intraparenchymal course and posterior-anterior electrode direction through occipital burr holes, disciplined stereotaxy is essential for stimulation of the appropriate target.

A 13-year-old female with a history of multifocal, independent, bitemporal-onset seizures since 12 months of age showing evidence of left-sided mesial temporal sclerosis on MRI, underwent placement of bilateral mesial temporal RNS leads. An O-arm spin was performed after the placement and the images obtained were fused to the preoperative CT images. It demonstrated curvature of the leads, with some deviation from the planned trajectory, but no deviation from the target, that was worse on the left side, compared to the right; the left lead was placed first, followed by the right lead. Following discussion with our epilepsy neurology colleagues in the operating room, electrophysiological measurements from the implanted leads showed cleared epileptic activity and therefore no repositioning was pursued. Our hypothesis at that time was that cerebrospinal fluid leakage distorted the underlying ventricular anatomy causing some curvature in the lead during transventricular course and prolonged consideration during surgery.

In conclusion, transventricular trajectories during RNS lead placement may lead to cerebrospinal fluid loss and associated lead deformation. The distal aspect of the lead may nonetheless reside in the desired surgical target. Neuromonitoring for epileptic signature can provide reassurance with regard to accurate lead placement, obviating the need for lead repositioning. Surgeons should also recognize that fused imaging may confuse inferred anatomic position from preoperative MRI with actual anatomy post brain shift.

## Introduction

It has been estimated that up to one-third of patients with epilepsy have medically refractory seizures [1]. Several surgical treatment options exist for this patient population which can be broadly divided into surgical resection and neurostimulation approaches, including vagus nerve stimulation (VNS), responsive neurostimulation (RNS, NeuroPace, Mountain View, CA), deep brain stimulation (DBS) and chronic subthreshold cortical stimulation [2-3]. In particular, RNS has become increasingly popular following a publication demonstrating its efficacy in a pivotal trial in 2014 [4]. RNS is most commonly indicated for patients with disabling focal-onset seizures secondary to bifocal-onset or a seizure focus in an unresectable location [4].

The RNS system is composed of an implanted cranial programmable neurostimulator connected to depth or subdural cortical strip leads [4]. In bilateral mesial temporal epilepsy cases, the electrodes are typically positioned under stereotactic guidance and passed in a posterior-anterior direction through occipital burr holes [5]. Given their long intraparenchymal course and magnification of small errors due to long distances, meticulous caution must be paid to ensure a straight and accurate trajectory to the surgical target. Here, we present a case of post-placement lead deformation secondary to cerebrospinal fluid (CSF) loss in transventricular trajectory.

## Technical report

**Figure 1:**
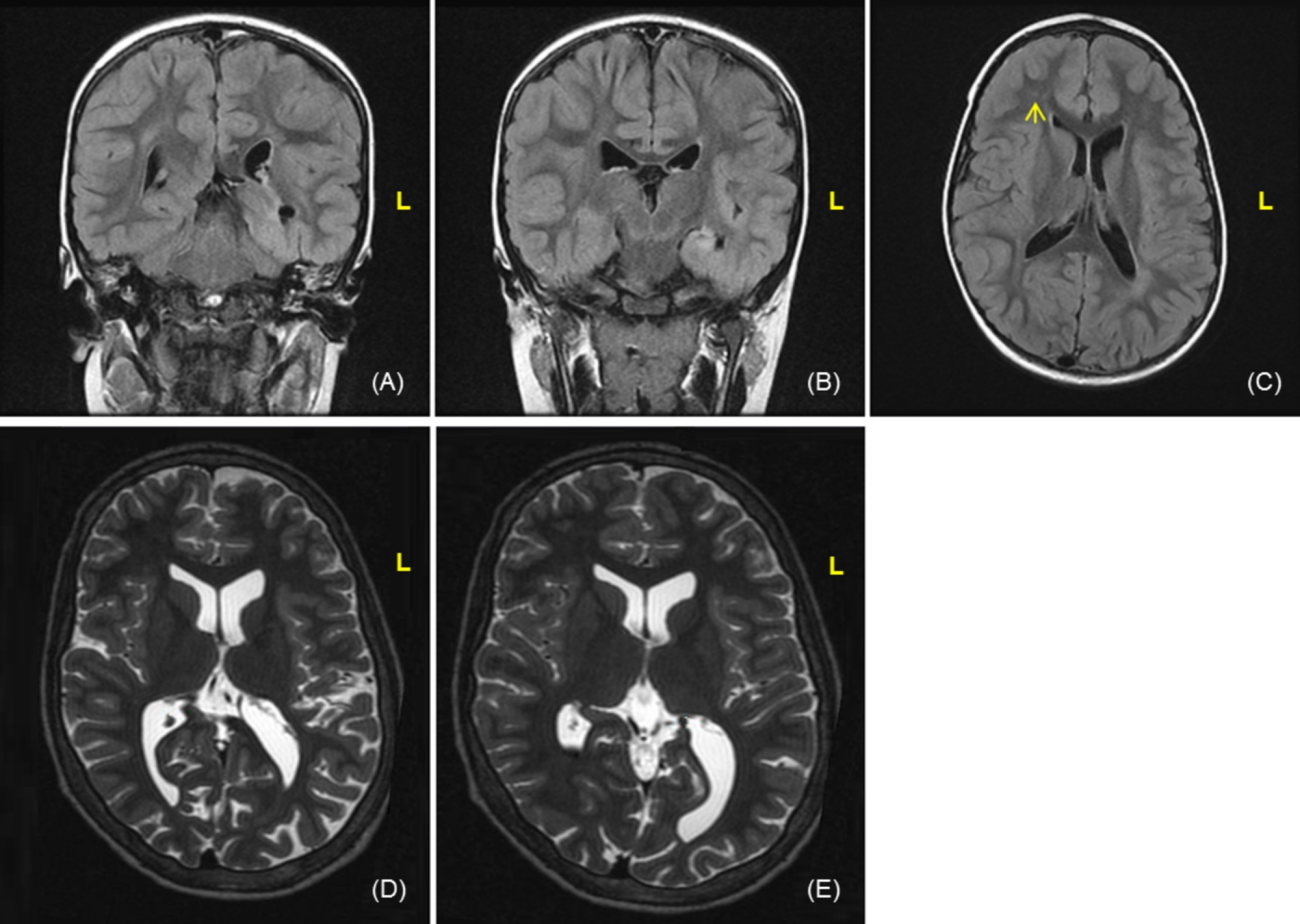
Magnetic resonance imaging of the brain Magnetic resonance imaging of the brain (A-B). There is evidence of abnormal T2 hyperintensity within the left hippocampus and mesial left temporal lobe and mild atrophy of the temporal portion of the left lateral ventricle, which are findings suggestive of mesial temporal sclerosis. In addition, there is evidence of a small focal nonspecific area of T2 hyperintensity seen within the right frontal white matter, which could reflect residua of prior infectious or inflammatory disease or related to seizure activity (yellow arrow) (C). There was no evidence to suggest right-sided mesial temporal sclerosis. There is also congenital enlargement of the occipital horns (D-E).

The patient is a 13-year-old right-handed female who presented with drug-resistant seizures since the age of 12 months. Her initial seizure was a prolonged, right-sided hemiclonic event, which was quickly followed by focal, non-motor, impaired awareness seizures characterized by staring, behavioral arrest, manual automatisms, chewing and right-or-left gaze deviation. The seizures occasionally evolved to hemiclonic or bilateral tonic-clonic activity. Her past medical history is significant for moderate intellectual disability (full-scale IQ 46) and attention deficit and hyperactivity disorder. The preoperative MRI demonstrated abnormal T2 hyperintensity within the left hippocampus and mesial left temporal lobe as well as mild atrophy of the temporal portion of the left lateral ventricle, which are findings suggestive of mesial temporal sclerosis. There was also evidence of a small focal nonspecific area of T2 hyperintensity seen within the right frontal white matter and congenital ventriculomegaly of occipital horns (Figure 1). There was no evidence of right-sided mesial temporal sclerosis. 

On electroencephalography, she was noted to have epileptiform discharges over both temporal regions, left greater than right. Interestingly, during continuous scalp recording, she was noted to have two of her typical focal-onset seizures originating from right temporal region. Her seizures had been extremely difficult to control despite her taking multiple antiepileptic medications and undergoing vagus nerve stimulation. Following evaluation by a multidisciplinary panel, the consensus decision was made to proceed with RNS lead implantation in bilateral hippocampi and amygdalae. 

**Figure 2:**
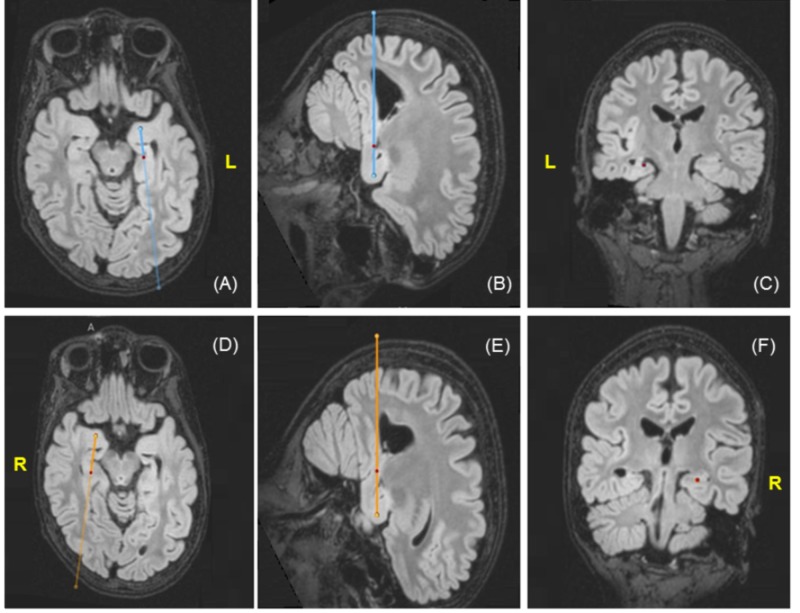
Stealth magnetic resonance images demonstrating the planned trajectory for the left-sided (A-C) and right-sided (D-F) leads The red circles represent the hippocampal target (D-F).

On the day of surgery, a stereotactic head frame was applied and a stereotactic head CT venogram was obtained to assist with surgical planning according to our institutional protocol, which was subsequently merged with the preoperative MRI (Figure 2).

**Figure 3:**
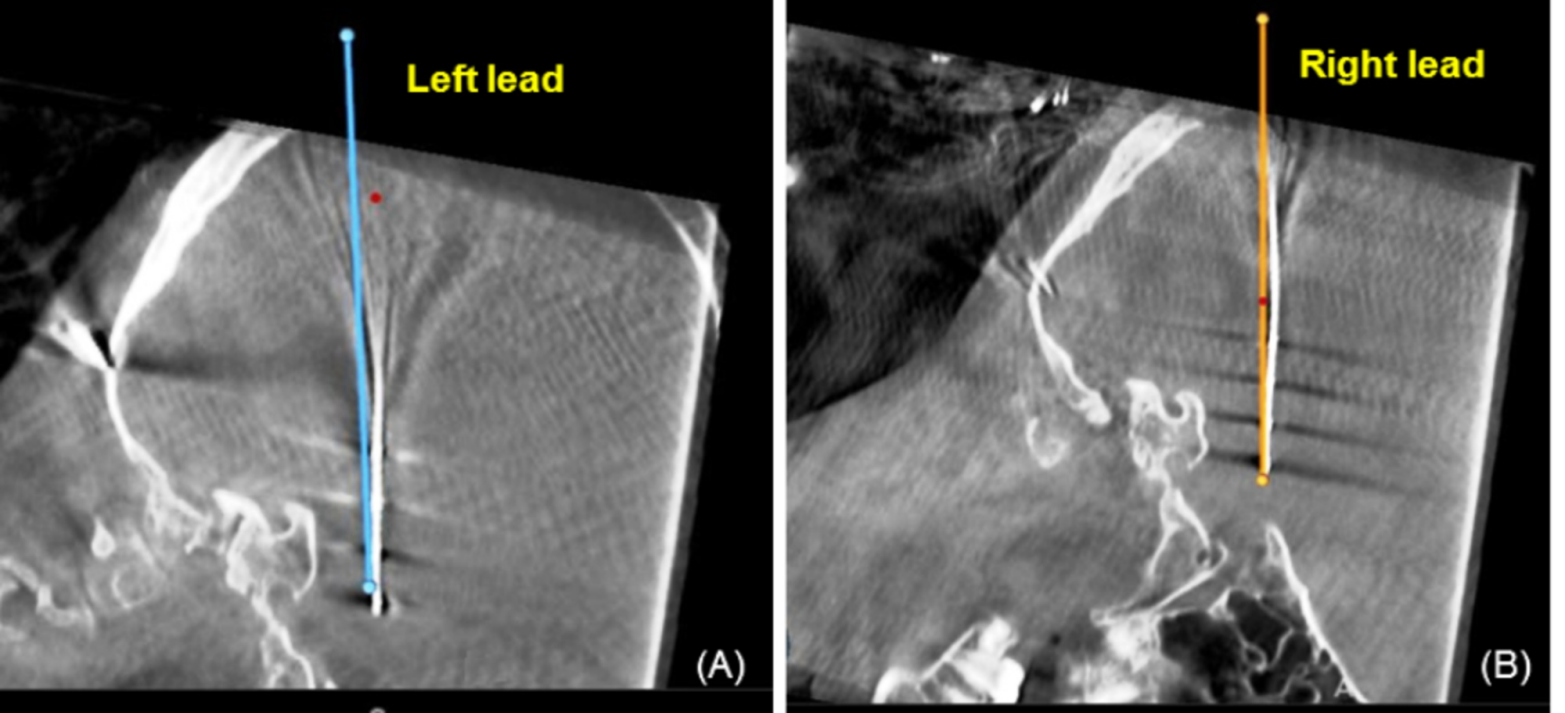
Intraoperative O-arm pictures demonstrating the lead deviation from planned trajectories, left (A) worse than right (B)

Leads were advanced to surgical target with stereo-navigation and a rigid cannula. Due to congenital enlargement of the occipital horns, there was no planned safe trajectory that could avoid a transventricular passage. Following placement of both leads, intraopeative O-arm (Medtronic, Dublin, Ireland) 3D pictures were obtained to verify placement and were fused to the preoperative imaging. These demonstrated local curvature and concern for deviation from the planned trajectory, left being worse than right (Figure 3).

**Figure 4:**
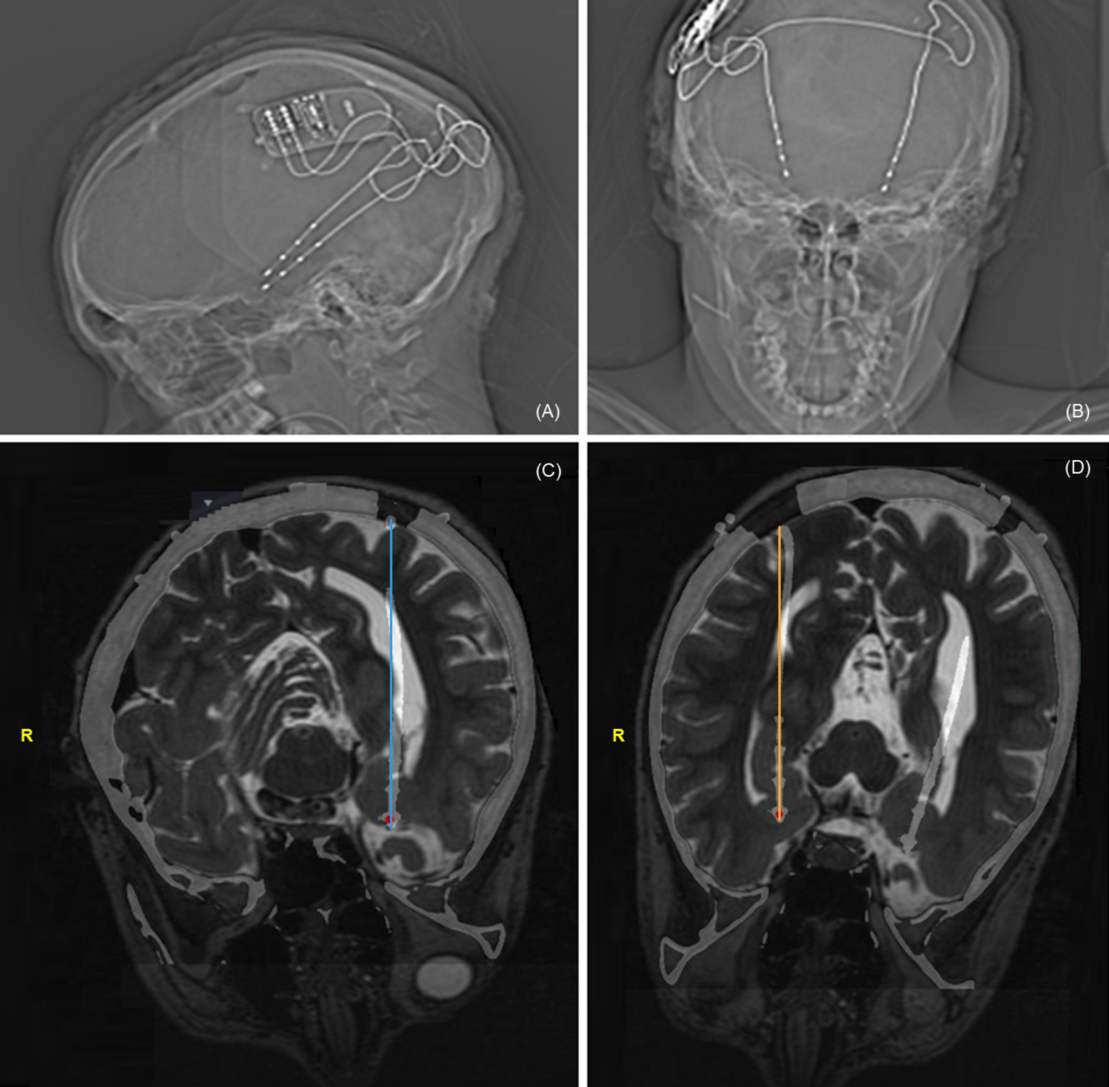
Postoperative X-rays (A-B) and merged CT-MRI (C-D) Postoperative X-rays (A-B) and merged CT-MRI (C-D) demonstrating the final lead position in bilateral mesial temporal structures (C-left, D-right) with associated curvature during intraventricular course.

This curvature coincided with the lead entry into the lateral ventricle, followed by straightening of trajectory where they re-entered the brain tissue. After consulting with the neurology epilepsy monitoring team intraoperatively, clear epileptic activity measured from the implanted RNS was confirmed and based upon this, the decision was made to leave them in place. Our hypothesis at that time was that CSF leakage following cannula insertion distorted the underlying anatomy causing some curvature in the lead and possible deformation, but the stimulating electrodes were actually in the intended tissue. This belief was later validated in the postoperative CT (merged with preoperative MRI), which showed the leads in the hippocampi and amygdalae (Figure 4). 

## Discussion

Hardware complications in functional neurosurgery are not uncommon; lead-related problems comprise the second most common complication following surgical site infections, with an estimated incidence of 3.8% [6]. They most commonly involve electrode breakage or migration, stimulator migration/malfunction, and skin erosion or infection. The complication rate per electrode-year is reported to be approximately 4.3% [7]. Lead deviations from planned trajectory or lead deformation are extremely rare events, especially with the advent of advanced neuro-imaging techniques that aid in optimizing lead placement into the surgical target.

The majority of evidence regarding the inaccuracy of anatomical targeting during neurostimulation is derived from DBS literature. In the RNS publication of long-term outcomes, there was no mention of complications related to transventricular trajectory; “device lead damage” occurred in 3.5% [8]. Furthermore, according to a later review by Sun and Morrell, four subjects had lead revisions in order to improve the lead location, however, further details were not provided [9]. Joint et al. first reported a case of two DBS leads transgressing the ventricles in a patient with ventriculomegaly [10]. They cited the lack of rigidity in Medtronic electrodes (3389 and 3387) in combination with fluctuation in resistance during passage leading to increased susceptibility to deviation and subsequent misplacement [10]. In our case, accurate lead positions were confirmed by verifying the epileptiform nature of recorded brain waves.

Zrinzo et al. later reported a 42% rate of transventricular electrodes in a cohort of 109 DBS patients [11]. Involving the ventricles resulted in a significantly higher targeting error rate when the electrode transgressed the ventricle (1.9±1.1 mm), with 19% of electrodes requiring multiple passes before final implantation. This error was attributed to the rigidity of the ventricular wall and CSF loss with subsequent brain shift. From a safety perspective, it should be noted that transventricular approaches have been previously associated with altered mental status in 1.2%, seizures in 0.6% and intraventricular hemorrhage in 0.5% (which was clinically asymptomatic) [12]. In a similar fashion, Elias et al. identified a total of 15 adverse events in 113 transventricular lead placements though none of these complications were directly linked to ventricular punctures [13]. The authors also reported a 5% rate of clinically asymptomatic intraventricular hemorrhage. Fortunately, our patient experienced no perioperative complications, was discharged home the next day and later showed a reduction in clinical seizures at the three-month follow-up. Counterintuitively, the first lead in such situations is likely the better-placed lead despite more curvature on imaging. This is because CSF leak occurs between the two placements and the rigid pass for the second lead is made through more distorted anatomy. 

## Conclusions

Lead placement during deep brain or responsive nerve stimulation procedures can be distorted during transventricular trajectories to the surgical target. Neurophysiological recordings can provide reassurance with regard to accurate lead placement and prevent unnecessary and possibly counterproductive lead repositioning.
